# The Theory of Planned Behavior as Applied to Preoperative Smoking Abstinence

**DOI:** 10.1371/journal.pone.0103064

**Published:** 2014-07-24

**Authors:** Yu Shi, Shawna Ehlers, David O. Warner

**Affiliations:** 1 Department of Anesthesiology, Mayo Clinic, Rochester, Minnesota, United States of America; 2 Department of Psychiatry and Psychology, Mayo Clinic, Rochester, Minnesota, United States of America; University of Bath, United Kingdom

## Abstract

Abstinence from smoking on the morning of surgery may improve outcomes. This study examined the explicatory power of the Theory of Planned Behavior (TPB) to predict smoking behavior on the morning of surgery, testing the hypothesis that the constructs of attitude, subjective norm, and perceived behavioral control (PBC) will predict intent to abstain from smoking the morning of surgery, and that intent will predict behavior. TPB constructs were assessed in 169 pre-surgical patients. Smoking behavior on the morning of surgery was assessed by self-report and CO monitoring. Correlations and structural equation modeling (SEM) were used to determine associations between measures and behavior. All TPB measures, including intent as predicted by the TPB, were correlated with both a lower rate of self-reported smoking on the morning of surgery and lower CO levels. The SEM showed a good fit to the data. In the SEM, attitude and PBC, but not subjective norm, were significantly associated with intent to abstain, explaining 46% of variance. The effect of PBC on CO levels was partially mediated by intent. The amount of variance in behavior explained by these TPB constructs was modest (10% for CO levels). Thus, attitude and perceived behavioral control explain a substantial portion of the intent to maintain preoperative abstinence on the morning of elective surgery, and intent and perceived behavioral control explain a more modest but significant amount of the variance in actual smoking behavior.

***Trial Registration***: Clinical Trials.gov registration: NCT01014455

## Introduction

Abstinence from smoking quickly decreases the levels of smoke constituents such as carbon monoxide (CO) and nicotine in the body because the half-life of these compounds is relatively short (approximately 4 and 1 hours, respectively) [1]. These and other constituents may have adverse effects in patients undergoing surgery, and current recommendations state that cigarette smokers should abstain from smoking for at least 12 hours prior to surgery [1]. However, many continue to smoke on the morning of surgery, either because clinicians do not provide this advice or because patients are not adherent [2], [3].

**Figure 1:**
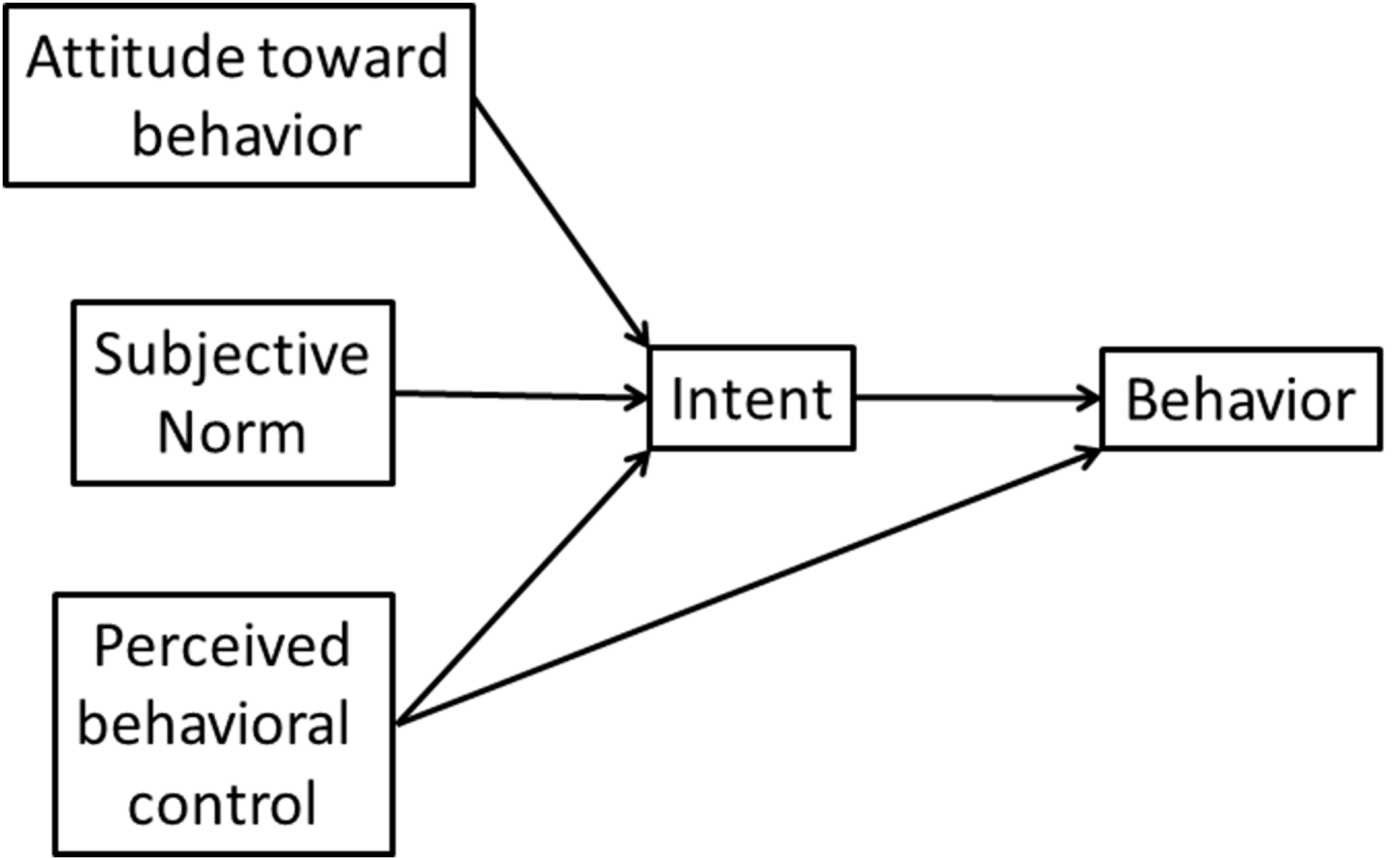
Theory of Planned Behavior.

A prior report described a randomized trial testing the hypothesis that pre-surgical patients would be more likely to comply with advice not to smoke the morning of surgery if they were also informed that exhaled CO levels would be assessed on the morning of surgery to assess adherence [4]. The theoretical framework underlying this hypothesis was conceptualized in terms of the Theory of Planned Behavior (TPB) [5], which many studies have evaluated in the context of smoking behavior [6] (Figure 1). We speculated that informing the subject about preoperative CO monitoring would reinforce the importance of normative beliefs regarding the importance placed on preoperative abstinence by their clinician, and increase their motivation to comply with advice to abstain. However, the results of this trial did not support the hypothesis. Although those informed of the CO monitoring did express a greater intent to maintain abstinence, they were not more likely to abstain from smoking the morning of surgery [4].

As a part of this clinical trial, measures of TPB constructs were tailored for the pre-surgical setting and administered as an *a priori* plan to determine potential mediators of treatment effect based on the predictions of the TPB [4]. As no treatment effect was observed, these measures were not presented in the prior report. Nonetheless, these measures provide an opportunity to examine the explicatory power of the TPB in the context of preoperative smoking, which is the objective of the current report. Specifically, we hypothesized that the constructs of attitude, subjective norm, and perceived behavioral control would be associated with intent to abstain from smoking the morning of elective surgery, and that intent and perceived behavioral control would be associated with actual behavior, as assessed by the exhaled CO level the morning of surgery and self-report of smoking on the morning of surgery. A better understanding of the factors determining preoperative smoking behavior may aid in the future design of interventions to promote preoperative abstinence.

## Materials and Methods

### Ethics statement

This study was reviewed and approved by the Mayo Clinic Institutional Review Board, Rochester, Minnesota, and written informed consent was obtained from all subjects. All clinical investigation was conducted according to the principles expressed in the Declaration of Helsinki.

### Setting and subjects

Study procedures have been previously described in detail [4]. To summarize, subjects scheduled for elective surgery were recruited from the Mayo Clinic Rochester Pre-Operative Evaluation Center. Eligibility criteria included age ≥18 years and current smoking at the time of evaluation, defined as >100 cigarettes lifetime consumption [7] and self-report of smoking either every day or some days. Exclusion criteria included an inability to understand consent procedures or inability to complete a written questionnaire.

### Procedure

Study subjects were randomized to one of two conditions. All study subjects received advice recommending preoperative abstinence, including the rationale for doing so (e.g., reducing the risk of perioperative complications). Subjects randomized to the CO-informed condition were told that their smoking status would be checked by exhaled CO monitoring on the morning of surgery, and why this was important. Subjects randomized to the control condition received a general stop-smoking message (not specifically related to preoperative abstinence) to ensure that the time spent with subjects was comparable between the two groups. Subjects were then administered a survey instrument (described in the following section) and discharged from the preoperative center.

Study personnel attended the pre-surgical admission of subjects who had undergone these study procedures in the preoperative center. This admission process includes measurement of exhaled CO levels (Micro Smokerlyzer; Bedfont, United Kingdom) and a brief recent smoking history, including whether they had smoked a cigarette the morning of surgery. Informed consent was obtained after admission (a procedure approved by the Institutional Review Board), because informing patients of the study at time of enrollment would have required providing information making it impossible to evaluate the hypothesis. Study records from patients who declined informed consent (n = 14) were destroyed and are not included in the analysis.

### Research assessments

We tailored an instrument to measure the constructs of the TPB (attitudes, subjective norm, perceived behavioral control, and intent) related to smoking behavior in the preoperative period according to the manual of Francis et al [8], building upon our prior formative research with cigarette smokers undergoing surgery [9]. The instrument included items querying 1) attitudes towards smoking the morning of surgery (e.g., “Not smoking the morning of surgery will be beneficial to me”); 2) subjective norm related to physicians (e.g., “My doctors think I should not smoke the morning of surgery”); 3) perceived behavioral control (PBC) (e.g. “If I wanted to, I would be able to stay off cigarettes the morning of surgery”), and; 4) intent (e.g., “I plan not to smoke the morning of surgery”). Three to four items were tailored to directly measure each construct. Indirect measures of TPB constructs were not included to minimize respondent burden in this busy preoperative clinic. Seven point Likert scales were utilized in the responses to all items and scored consistent with the manual [8]. The instrument was piloted with 5 patients who were eligible for the study, and their feedback was incorporated in a final version of the questionnaire (see Appendix S1). This TPB instrument was combined with baseline smoking history including the Fagerstrom Test for Nicotine Dependence (FTND) [10] to form the questionnaire that was administered in the preoperative center immediately after interventions were delivered by the study personnel.

### Analysis

For the analysis of the items used to measure TPB constructs, their internal consistency was assessed by calculating Cronbach’s coefficient alpha [11]. To determine the impact of each of the individual items on the scale’s internal consistency, the alpha was recalculated after deletion of each individual item. If substantial improvements in reliability were obtained, the item was removed. Alpha levels of 0.6 or higher were considered to be indicative of satisfactory internal consistency [12]. An average score of the remaining items was computed for each construct and utilized in further analysis. The factor structure of items measuring each of the constructs was characterized using principal component factor analysis of the candidate items.

As described in the Results, the primary outcomes of exhaled CO and self-reported abstinence on the morning of surgery did not differ between experimental groups [4]. Thus, for the present analysis data from these two arms were combined. Pair-wise Pearson’s product moment correlations between variables were first assessed by a correlation matrix. To evaluate the relevance of the TPB model (Figure 1) in the context of preoperative smoking behavior, structural equation modeling (SEM) was performed. A path analysis included attitude, subjective norm, perceived behavioral control, intent, and smoking behavior measured by CO levels. The SEM did not include any latent variable. Maximum likelihood method was used in estimation. Goodness of fit of the model was tested by chi-square test and root mean square error of approximation. The TPB posits a potential direct effect of perceived behavioral control on behavior (not mediated by intent). This possibility was evaluated by indirect and direct effects of perceived behavior control on smoking behavior in the SEM.

Analysis was performed using Stata, version 13.1 (College Station, TX), and *p*<0.05 was considered to be statistically significant.

## Results

From February 2010 to February 2011, 169 subjects were enrolled in the study and underwent surgery (Table 1). Among all subjects, 56 (33.1%) received outpatient surgery. The median time from study assessment at preoperative center to surgery was 1 day with an interquartile range of 1 to 3 days. As previously reported [4], on the morning of surgery CO levels and self-reported smoking behaviors were not significantly different between intervention groups; CO levels were 9.7±8.0 [M±SD] and 9.3±6.6 in in the CO-informed and control groups, respectively (p = 0.67), and 21% and 25% of subjects in the CO-informed and control groups, respectively, self-reported smoking the morning of surgery (p = 0.48). Thus, in subsequent analyses TPB constructs were analyzed by combining data from both experimental groups.

**Table 1:** Baseline characteristics of the study population (n = 169).

Age (M ± SD)	52.8±12.0
Sex (Male)	82 (48.5%)
Race (Caucasian)	151 (95.6%)
Occupation	
Employed	85 (53.8%)
Unemployed	39 (24.7%)
Retired	34 (21.5%)
Marital status	
Married	95 (56.9%)
Not married	66 (39.5%)
Widowed	6 (3.6%)
Education	
Less than high school	12 (7.6%)
High school (GED)	63 (39.9%)
Some college	56 (39.9%)
College or above	27 (35.4%)
Inpatient surgery	113 (66.9%)
Cigarettes per day (M±SD)	14.9±9.3
Previous quit attempts	
0	21 (12.5%)
1	39 (23.2%)
2–5	82 (48.8%)
6–10	14 (8.3%)
>10	12 (7.1%)
FTND	3.5±2.2

Continuous variables are reported as Mean ± SD. Categorical variables are reported as n (%). FTND, Fagerstrom test for nicotine dependence; GED, General educational development.

Regarding the properties of the TPB measures, the Cronbach’s alpha of the 4 items querying attitudes was 0.52 and increased to 0.70 after elimination of the item “not smoking the morning of my surgery will be unpleasant”. Factor analysis of the remaining 3 items suggested a single factor with Eigenvalue of 1.40. After varimax rotation, the loadings of the 3 items on the single factor were 0.80, 0.81, and 0.35. An average score of the three items was calculated and utilized as measurement of attitude in further analysis. The 3 items querying subjective norm had a Cronbach’s alpha of 0.23. Removal of the item “I feel pressure from my doctors not to smoke the morning of surgery” increased the alpha to 0.54. An average score of the 2 items was calculated and utilized as measurement of subjective norm. The Cronbach’s alpha of the 3 items querying perceived behavioral control (PBC) was 0.76. Factor analysis suggested a single factor with Eigenvalue of 1.46. After varimax rotation, the loadings of the 3 items on the single factor were 0.79, 0.66, and 0.63. An average score of these three items was calculated and utilized as measurement of PBC. The Cronbach’s alpha for the 3 items on intent was 0.57 and increased to 0.72 after removal of the question “How likely is it that you will not smoke cigarettes the morning of surgery?” An average score of the remaining 2 items was utilized as the measurement of intent in subsequent analyses.

Regarding attitudes, most subjects strongly agreed that not smoking the morning of surgery was beneficial (score for attitude of 2.7±0.6 on a Likert scale ranging from −3 to 3). Regarding subjective norm, most subjects strongly agreed that their doctors thought they should not smoke the morning of surgery (subjective norm score of 2.6±1.0, scale ranging from −3 to 3). Subjects also expressed a high degree of perceived behavioral control for being able to stay off cigarettes the morning of surgery if they wanted/decided to (mean score of 6.5±1.0, scale ranging from 1 to 7). Most subjects reported that they intended not to smoke the morning of surgery (intent score of 6.5±1.1, scale ranging from 1 to 7).

Table 2 displays the correlations between TPB measures. All TPB measures (attitude, subjective norm, perceived behavioral control, and intent) were significantly correlated with each other. Self-reported smoking was correlated well with CO levels (coefficient of 0.58, *p*<0.001) As predicted by the TPB model, intent was significantly correlated with both a lower rate of self-reported smoking on the morning of surgery (coefficient of −0.34, p<0.0001) and lower CO levels (correlation of −0.28, p = 0.0004).

**Table 2:** Correlations between TPB variables.

	Attitude	Subjectivenorm	Perceived behavioralcontrol	Intent
Attitude	1.0000			
Subjectivenorm	0.5704	1.0000		
Perceivedbehavioral control	0.6300	0.358	1.0000	

In the correlation matrix, the correlation is presented for each pair of variables, and below that correlation is The *P* value associated with the hypothesis test that the correlation is zero was <0.00001 for each comparison, with the exception of the correlation between perceived behavioral control and subjective norm, which was <0.0003. TPB, Theory of planned behavior.

**Figure 2:**
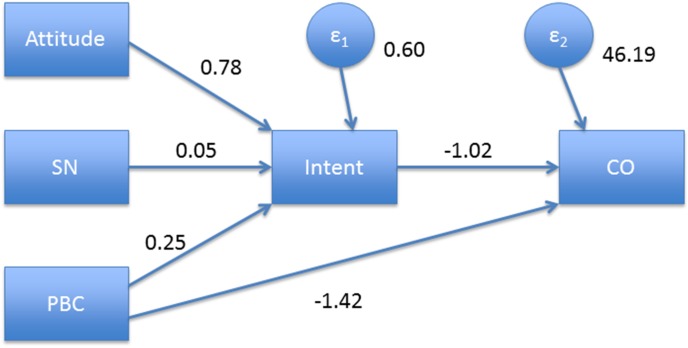
Path analytic model showing standardized path coefficients and error terms (E1 and E2). Abbreviations: SN, subjective norm; PBC, perceived behavior control; CO, carbon monoxide. Path coefficients and levels of statistical significance are: Attitude and intent, 0.78, *p*<0.001; SN and intent, 0.05, *p* = 0.52; PBC and intent, 0.25, *p* = 0.001, intent and CO, −1.02, *p* = 0.10; PBC and CO, −1.42, *p* = 0.027.

Figure 2 presents the SEM path analysis of the TPB model. The chi-square test indicated a good fit for the overall model (chi-square = 0.97, *p* = 0.617). The R^2^ for the model leading to intent was 0.46 while the R^2^ for the model including CO levels as the measure of smoking behavior was 0.097. The relationships between attitude and intent, PBC and intent, PBC and CO levels were statistically significant at *p*<0.05. In the SEM analysis, the path coefficient for the total effect of PBC on CO levels was −1.68, *p* = 0.004, with a path coefficient for the direct effect of −1.42, *p* = 0.027. The path coefficient for the indirect effect of PBC through intent was −0.26, *p* = 0.144. This result suggested that the effect of PBC on CO levels was primarily via a direct effect, consistent with the modest relationship between intent and CO levels (path coefficient of −1.02, p = 0.10).

## Discussion

The main finding of this study is that measurements of TPB constructs can explain a small but significant amount of the variance in preoperative smoking behavior on the morning of surgery.

It is well-established that elective surgery represents a “teachable moment” for postoperative abstinence, as quit rates are increased (compared with similar subjects not undergoing surgery) even in the absence of interventions [13], [14]. The mechanisms responsible for this finding are unknown. In terms of behavioral theories, various measures of intent and self-efficacy appear to be important predictors of postoperative abstinence [2], [15], but no behavioral theory has been previously evaluated in regards to perioperative smoking behavior (either before or after surgery). The importance of intent and self-efficacy to postoperative abstinence (the latter conceptualized as PBC) in the TPB suggest its possible application to the perioperative setting.

The data in the current report were generated as part of a previously-reported trial which examined whether informing preoperative patients that CO levels would be checked the morning of surgery would increase compliance with advice to not smoke the morning of surgery [4]. In terms of the TPB, we hypothesized that informing the subject would heighten normative beliefs regarding the importance of complying with clinician recommendations, and increase their intent to not smoke the morning of surgery. Although we noted a tendency for a higher subjective norm score in patients in the CO-informed condition, this did not reach statistical significance (data not shown). As previously reported [4], those informed of CO monitoring did express greater intent to maintain abstinence (*P* = 0.040), but this did not translate into a significant difference in behavior; there was no significant difference between experimental groups in either CO of self-reported smoking behavior the morning of surgery [4]. As discussed in the previous report, the control intervention itself was efficacious in promoting abstinence; informing patients about CO monitoring did not produce further changes in attitudes, PBC, and smoking behavior.

In our analysis, the measures of attitude, subjective norm, and PBC were individually all well-correlated with intent in univariate analysis, as predicted by the TPB model. However, in the SEM that included all three factors, subjective norm was no longer an independent predictor of intent. According to the TPB, subjective norm is defined as “belief about whether most people approve or disapprove of the behavior” [16]. Because belief about surgical providers’ norm was the target of the controlled trial, the items assessing this construct did not address other potential referents (family, friends, etc). Our results were consistent with previous studies in that subjective norm is less likely to be a significant factor predicting intent compared with attitude and self-efficacy [17]. This consideration may have contributed to the modest effect of the intervention, which was designed specifically to influence social norm, on intent. The three-factor model explained 46% of the variance of intent not to smoke the morning of surgery. According to a review of the TPB, this value compares favorably with a mean value of 41% in studies applying the TPB to a variety of other behaviors [17].

In contrast to its ability to predict intent, the ability of the TPB to predict actual behavior was modest, as only a small part of variance of CO levels on the morning of surgery was explained by the combination of intent and PBC. Indeed, although there were significant correlations between intent and behavior in univariate analysis, this relationship was not significant in SEM, with PBC having a significant direct effect on CO levels. Previous research on TPB shows that its ability to predict behavior varies depending on population and specific behavior [16]. For example, in one longitudinal study looking at smoking cessation outcomes in 3–4 months following initial assessment, intent only accounted for a small amount of the variance in behavior [18]. Despite the fact that in the current study the measurement of behavior happened in a short period after the initial assessment, the predictive ability was still modest. Nonetheless, given that PBC did directly affect CO levels, the results suggest that PBC could be a target in developing interventions to promote smoking cessation for surgical patients. For example, interventions could highlight the findings from our prior work that abstinence from smoking in the perioperative period is not associated with increases in perceived stress or cigarette cravings [3]; i.e., smokers may find it easier to quit in the perioperative period compared with other times. Tobacco control interventions targeting self-efficacy can be efficacious in increasing smoking cessation [19], [20], although it is not clear if smoking cessation was actually mediated by self-efficacy [19].

Limitations of this study include the relatively low internal consistency for the scale measuring subjective norm, which may have limited the ability to detect significant associations. Also, the study population was primarily Caucasian, well-educated, and drawn from a tertiary care center, which may limit generalizability. The number of subjects did not permit the evaluation of potential for factors such as the intensity of surgery (inpatient vs. outpatient), nicotine dependence, and others to moderate the effects of TPB measures on intent and behavior. Finally, there are a variety of other behavioral theories, including theories of threat or motivation, which could provide alternative explanations for intent and behavior in the context of this trial. Measurements and analysis of constructs relevant to these theories would be of interest in future work but are beyond the scope of the current study.

## Conclusions

The Theory of Planned Behavior constructs of attitude and perceived behavioral control explain a substantial portion of the intent to maintain preoperative abstinence on the morning of elective surgery. Intent and perceived behavioral control explain a more modest but significant amount of the variance in actual smoking behavior. This information may be useful in the future design of interventions to promote preoperative abstinence from smoking.

## Supporting Information

Appendix S1 (DOCX)
