# Supplementary material for: The Theory of Planned Behavior as Applied to Preoperative Smoking Abstinence
Source: PLoS One. 2014 Jul 24;9(7):e103064. doi: 10.1371/journal.pone.0103064 (PMC4109996; doi:10.1371/journal.pone.0103064)
Supplement: Appendix S1 — (DOCX) [file pone.0103064.s001.docx]

**Appendix S1 - Initial items used to measure Theory of Planned Behavior constructs**

Attitude

1. It would be good for me if I did not smoke the morning of my surgery.

Strongly disagree----Strongly agree

1. Not smoking the morning of my surgery will be unpleasant.

Strongly disagree----Strongly agree

1. Not smoking the morning of my surgery will be helpful to me.

Strongly disagree----Strongly agree

1. Not smoking the morning of my surgery will be beneficial to me.

Strongly disagree----Strongly agree

Subjective Norm

1. My doctors think I should not smoke the morning of surgery.

Strongly disagree----Strongly agree

1. My doctors would disapprove of my smoking the morning of surgery.

Strongly disagree----Strongly agree

1. I feel pressure from my doctors not to smoke the morning of surgery.

Strongly disagree----Strongly agree

Perceived Behavioral Control

1. If I wanted to, I would be able to stay off cigarettes the morning of surgery.

Strongly disagree----Strongly agree

1. If I decided not to smoke the morning of surgery, I would be likely to succeed. (

Unlikely----Likely

1. I am confident that I could not smoke the morning of surgery if decided not to.

Strongly disagree----Strongly agree

Intent

1. I plan not to smoke the morning of my surgery.

Strongly disagree----Strongly agree

1. I intend not to smoke the morning of my surgery.

Strongly disagree----Strongly agree

1. How likely is it that you will not smoke cigarettes the morning of surgery?

Unlikely----Likely
